# GC-MS Techniques Investigating Potential Biomarkers of Dying in the Last Weeks with Lung Cancer

**DOI:** 10.3390/ijms24021591

**Published:** 2023-01-13

**Authors:** Elinor A. Chapman, James Baker, Prashant Aggarwal, David M. Hughes, Amara C. Nwosu, Mark T. Boyd, Catriona R. Mayland, Stephen Mason, John Ellershaw, Chris S. Probert, Séamus Coyle

**Affiliations:** 1 School of Medical and Health Sciences, Bangor University, Bangor LL57 2DG, UK; 2 Institute of Systems Medicine and Integrative Biology, University of Liverpool, Liverpool L69 7BE, UK; 3 School of Medicine, Cedar House, University of Liverpool, Liverpool L69 3GE, UK; 4 Department of Health Data Science, University of Liverpool, Liverpool L69 3GF, UK; 5 Academic Palliative & End of Life Care Department, Liverpool University Hospitals NHS Foundation Trust, Liverpool L7 8XP, UK; 6 Lancaster Medical School, Lancaster University, Lancaster LA1 4YW, UK; 7 Marie Curie Hospice Liverpool, Liverpool L25 8QA, UK; 8 Liverpool Head and Neck Cancer Centre, University of Liverpool, Liverpool L69 3BX, UK; 9 Department of Oncology and Metabolism, University of Sheffield, Sheffield S10 2RX, UK; 10 Palliative Care Unit, Institute of Life Course & Medical Sciences, University of Liverpool, Liverpool L7 8TX, UK; 11 Department of Palliative Medicine, Clatterbridge Cancer Centre, Liverpool L7 8YA, UK

**Keywords:** GC-MS, lung cancer, dying, urine, VOCs, volatile, biomarkers, palliative, SPME

## Abstract

Predicting when a patient with advanced cancer is dying is a challenge and currently no prognostic test is available. We hypothesised that a dying process from cancer is associated with metabolic changes and specifically with changes in volatile organic compounds (VOCs). We analysed urine from patients with lung cancer in the last weeks of life by headspace gas chromatography mass spectrometry. Urine was acidified or alkalinised before analysis. VOC changes in the last weeks of life were identified using univariate, multivariate and linear regression analysis; 12 VOCs increased (11 from the acid dataset, 2 from the alkali dataset) and 25 VOCs decreased (23 from the acid dataset and 3 from the alkali dataset). A Cox Lasso prediction model using 8 VOCs predicted dying with an AUC of 0.77, 0.78 and 0.85 at 30, 20 and 10 days and stratified patients into a low (median 10 days), medium (median 50 days) or high risk of survival. Our data supports the hypothesis there are specific metabolic changes associated with the dying. The VOCs identified are potential biomarkers of dying in lung cancer and could be used as a tool to provide additional prognostic information to inform expert clinician judgement and subsequent decision making.

## 1. Introduction

Nearly 10 million people worldwide died from cancer in 2020; lung cancer, with the highest mortality, was responsible for 1.8 million deaths [1]. Predicting when a patient with advanced cancer is likely to die is a challenge and currently no prognostic test is available [2]. Little is known about the biochemical pathways that change as people die with cancer. A systematic review of biomarkers associated with dying identified common themes in cancer patients, irrespective of the type of malignancy. These included raised inflammatory markers (for example, C reactive protein), organ dysfunction (kidney, liver) and cachexia [3]. Given the common features shared in patients dying from cancer, a “dying process” has been proposed [3,4] but has not yet been described. Several validated prognostic tools aim to predict survival of patients with advanced cancer [5]. A recent comparison of five validated prognostic tools showed the best were only as accurate as expert clinician judgement; the overall accuracy was 61% [6,7].

Metabolomics identifies de novo or changing metabolites, such as volatile organic compounds (VOCs) from biological samples [8,9]. VOCs are produced by a variety of processes including degradation from metabolic pathway intermediates; therefore, their concentration in samples can give an insight into biochemical activity upstream. They also have the potential to be biomarkers of disease [10,11]. A variety of biofluids are amenable to VOC analysis; urine is ideal as it can be sampled frequently, easily, non-invasively and stored for long periods [12]. Additionally, urine samples generate an intricate and comprehensive metabolomic profile, with VOCs ranging in polarity and complexity [13]. An added benefit of using urine is that it contains end products from multiple metabolic processes.

Urinary VOCs have been used to study both healthy and diseased states [14]. A recent review revealed 444 VOCs associated with urine [14]. VOCs in urine cover a range of chemical classes, e.g., acids, alcohols, ketones, aldehydes, amines, N-heterocycles, O-heterocycles, sulphur compounds and hydrocarbons [14]. The exact origins of VOCs are not always clear. However, ketones are highly prevalent in urine, composing approximately 20% of all urine [15,16,17]. Ketones are considered to be by-products of lipid metabolism [14]. Terpenes are considered to be derived from food [14]. Phenol and 4-methylphenol have been reported to increase in urine with increasing protein intake [14]. Recently, a study has showed 299 compounds of volatile/semi-volatile compounds could be generated by oxidation of a range of unsaturated fatty acids [18].

SPME is a solvent-less method that has been in use for over 30 years. It relies on the mass transfer between the sample and the headspace then the headspace and the fibre. The fibre is then injected in a gas chromatography instrument to separate and identify the compounds extracted. SPME’s main advantages are that samples do not require manipulation prior to use and it is fast [19]. This may occur in two different ways: headspace (HS-SPME) or direct immersion (DI-SPME). In HS-SPME, the fibre is exposed in the vapour phase above a gaseous, liquid or solid sample [19]. In our case, we utilised HS-SPME. Headspace (HS) solid phase microextraction (SPME) fibres adsorb VOCs in the headspace (HS) of a sample—improving on gas chromatography–mass spectrometry (GC-MS) alone. HS-SPME-GC-MS is a fast and cost-efficient technique that identifies numerous VOCs in biological material [20].

Urinary VOCs can aid detection a range of conditions, in particular cancer [21]. Several studies have examined urinary VOCs using SPME GC-MS. Our research group has recently shown that urinary VOCs can support the diagnosis of prostate and bladder cancer [22,23]. We recently described optimised protocols for the detection of VOCs using HS-SPME-GC-MS in human urine [24].

In this study, we hypothesised that a dying process from cancer is associated with metabolic changes and specifically with changes in volatile organic compounds. To test this, we collected urine from patients with lung cancer, the most common cancer in our region, in the last weeks of life. We then investigated changes in VOCs by HS-SPME-GC-MS for potential biomarkers of dying.

## 2. Results

We previously demonstrated that different VOCs are detected by acid and alkali treatment urine by HS-SPME-GC-MS [24]. We therefore used both techniques to discover potential biomarkers of dying. The acid dataset comprised 144 samples (55 samples in the last 3 weeks, 25 in last week of life) and the alkali dataset comprised 116 (43 samples in the last 3 weeks, 17 in the last week of life). More acid samples were run, due to limited sample volume obtained from some patients. The clinical characteristics of the patients are described in Table 1.

### 2.1. VOCs Changed toward Death in Acid-Treated Urine Dataset

After removing sparse data, 82 VOCs remained in the acid dataset (Table S1). As detailed in the methods, missing values were imputed, and the data was glog transformed (Table S2).

Since VOCs were detected in a semi-quantitative manner, VOC datasets were analysed as quantitative continuous variables. In the acid dataset, excluding NAs (where VOCs were not detected), the quantification of VOCs ranged from 2.44 × 10^5^ to 2.42 × 10^9^. This demonstrates that there are 1 × 10^4^-fold differences between the lowest detected VOC and the most abundant VOC detected. The coefficient of variation (CV) of VOCs was between 8% and 104% in pooled samples (Table S3). CV is not reported for non-pooled samples as abundance varied towards death.

In order to investigate changes in VOCs for potential biomarkers of dying, we analysed the same data in a number of ways and used a number of statistical methods, all with the same goal. Firstly, after dividing the samples into two groups, those samples closer to death and those further away from death, the VOCs between these groups were compared by fold change and t-tests (univariate analysis). Subsequently dividing the samples into five groups (week categories) allowed us to identify VOCs that changed over time towards death. Finally, linear regression analysis of the same data censored at different time points allowed us to find VOCs which had a clear trend towards death. It was therefore anticipated that these different approaches should find the same VOCs that change towards death.

Univariate analysis of the acid dataset identified 16 VOCs (out of 82, 20%) that changed significantly between groups, “days 0–21” versus “days 22+”. Nine VOCs increased towards death and a further two approached significance (*p* < 0.06, BH adjusted). Seven VOCs decreased towards death (Table 2). Data were visualised by Volcano plot (Figure S1). The acid dataset was also analysed by other boundaries of time before death (i.e., “day 0–7” versus “day 8+” and “day 0–15” versus “day, 16+”); this did not change the VOCs identified (Table S4).

Multivariate analysis performed on the acid dataset via week categories identified 17 VOCs as statistically significant (Table S5). A total of 4 VOCs increased towards death, 11 decreased and 2 were significant without a clear trend towards death.

We used PLS-DA to examine if the acid dataset could be classified by week categories, whilst the data showed a clear trend from week 1 (red) to week 2 (yellow), week 3 (green) and week 04+ and week 12+ (blue and purple) (Figure S2). The cross validation of this model yielded relatively low R2 and Q2 values of 0.43 and 0.27. Generally speaking, an R2 value above 0.7 would be considered a substantial predictive ability. Similarly, the Q2 should be close to the R2 value, indicating that the data fit the model. However, R2 and Q2 values were low.

Linear regression analysis of the acid dataset where time was censored at 21 days before death showed that 12 VOCs increased towards death and 22 decreased towards death (Table 2). Linear regression of the acid dataset was also undertaken by censoring at different timepoints before death (days 3, 7, 10, 15, 28 and 100) (Table S6).

A combination of univariate, linear regression and multivariate analysis of the identified VOCs showed that 14 VOCs increased and 23 VOCs decreased in the last weeks of life (Table 2).

**Figure 1:**
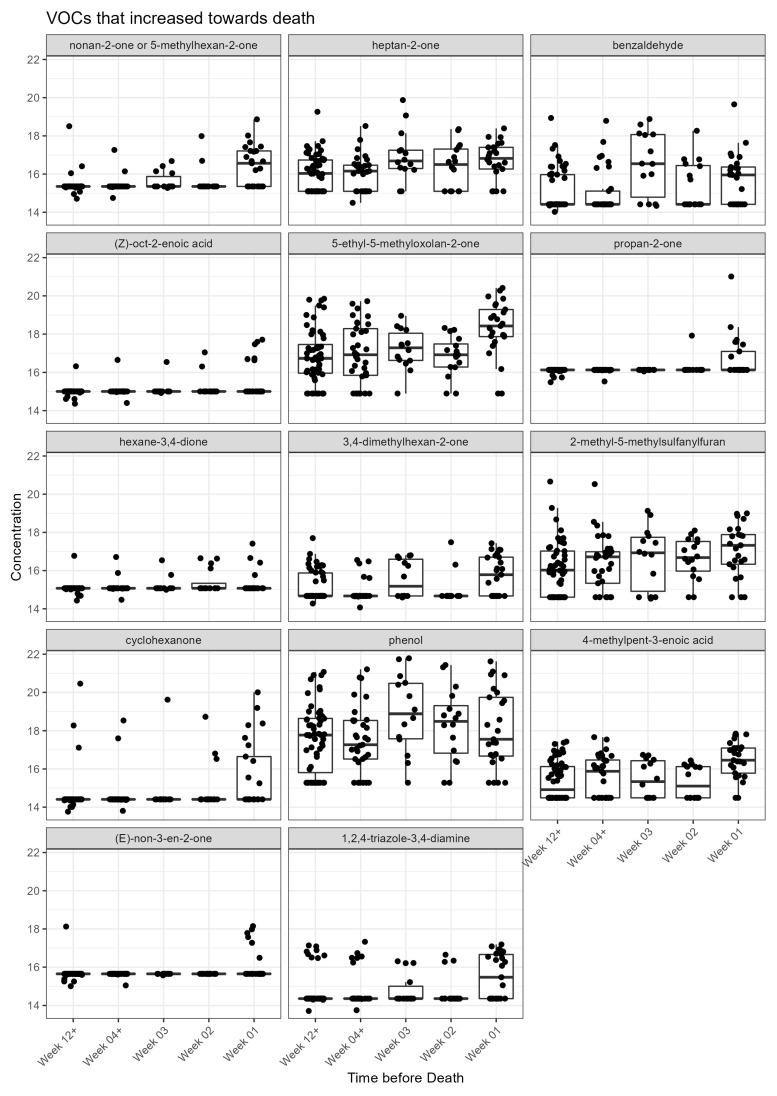
VOCs that increase towards death in the acid dataset. Jitter box plot graph for each metabolite identified as significant from univariate and linear regression analysis. The centre line is the median; box limits are upper and lower quartiles; whiskers are 1.5× interquartile range; points are individual observations. *Y* axis is VOC concentration on glog scale.

**Figure 2:**
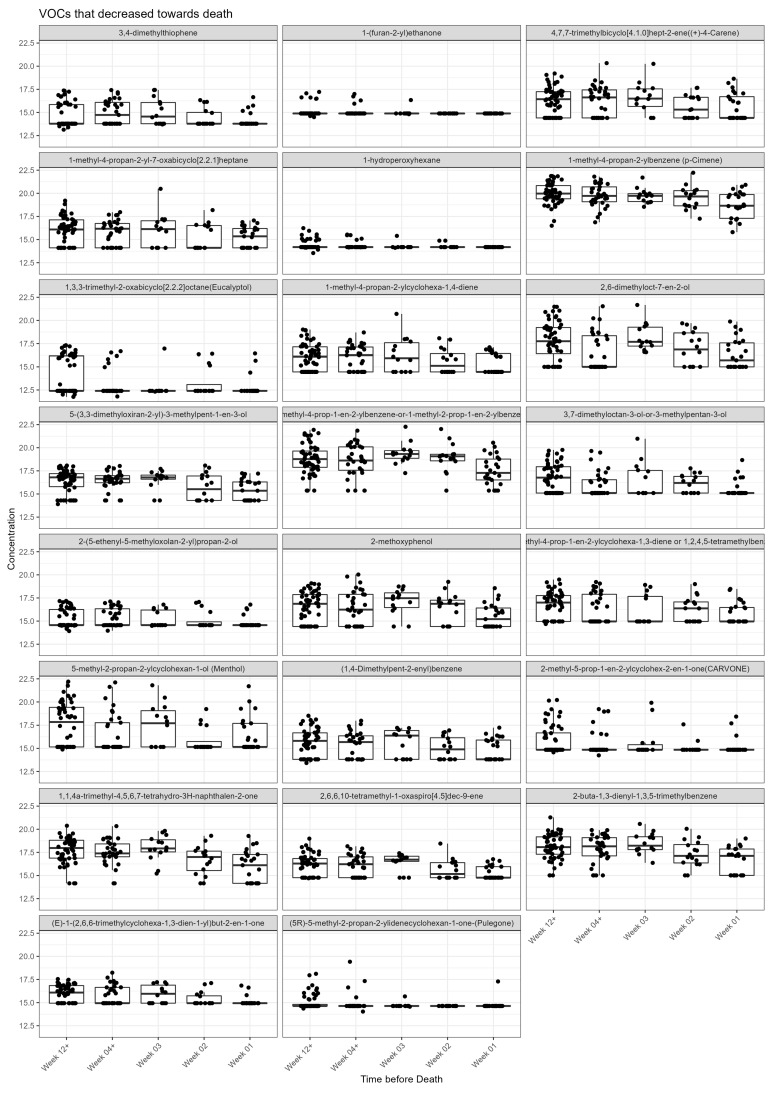
VOCs that decrease towards death in the acid dataset. Jitter box plot graph for each metabolite identified as significant from univariate and linear regression analysis. The centre line is the median; box limits are upper and lower quartiles; whiskers are 1.5× interquartile range; points are individual observations. *Y* axis is VOC concentration on glog scale.

Visual inspection of the box plots, for each VOC, by week categories towards death showed 11 increased towards death (Figure 1), whereas 23 decreased towards death (Figure 2). A total of 3 VOCs in the acid dataset found to be significantly increased towards death did not have a clear trend towards death (Figure 1: benzaldehyde, 3,4-dimethylhexan-2-one and phenol: two of these had larger CV >40%).

In the acidified set, of the 14 VOCs that increased towards death, 6 VOCs were ketones (Figure S3A). Of the 23 VOCs that decreased, 8 were unclassified by either MeSH or ChEBI; 6 were hydrocarbons; 3 were alcohols; 2 benzene derivatives; 2 heterocyclic compounds; 1 sulphur and 1 cosmetic (Figure S3B).

**Figure 3:**
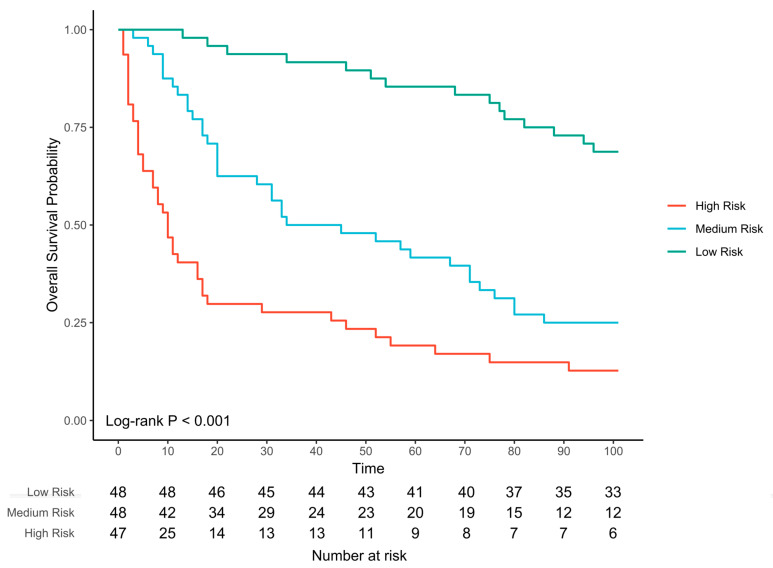
Kaplan–Meier survival curves using the Cox Lasso regression model. It shows three groupings based on prediction of 21-day risk, representing high, medium and low risk of dying.

**Figure 4:**
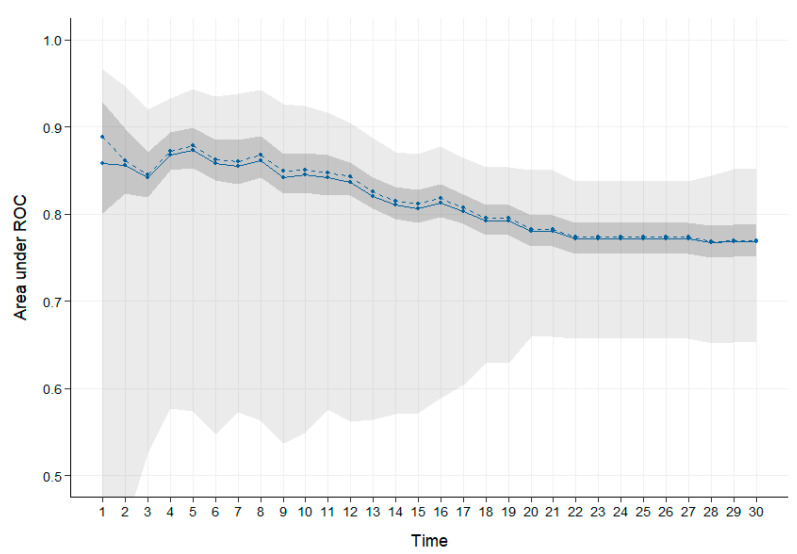
AUC for the Cox Lasso regression model. The blue line shows the mean and dotted line the median. The dark grey shows the confidence interval, and the light grey shows the minimum and maximum values.

We wanted to create a model to predict time to death that used VOC abundance. Cox models are commonly used to assess factors associated with time to death [25].The abundance of all VOCs are considered as possible predictors, and the lasso approach identifies the VOCs most strongly associated with time to death. The model utilised eight VOCs (Table 3). All of these VOCs had previously been identified by the other methods. Kaplan–Meier survival curves were plotted for patients classified as low, medium and high risk of dying (see Figure 3, log rank test *p* < 0.001). The high-risk group median survival is 10 days, the medium-risk group median survival is 50 days and low-risk group do not reach median survival. The model has excellent AUC values for every day in the last 30 days (0.77 at day 30, 0.78 at day 20 and 0.85 at day 10) (see Figure 4, Table S7). Calibration of the model was good (Figure S4). We visualised these eight VOCs by box plot over time towards death in week categories (Figure S5).

### 2.2. VOCs Changed toward Death in Alkali-Treated Urine Dataset

After removing sparse data, 33 VOCs were detected in the alkali dataset (Table S8); 18 of these VOCs were shared by the acid dataset. As detailed in the methods, missing values were imputed and the data was glog transformed (Table S9). Our analysis approach of the alkali dataset matched that of the acid dataset, where univariate, multivariate and linear regression were used to identify VOCs that change towards death. The coefficient of variation (CV) of VOCs in the alkali pooled samples was between 14% and 64% (Table S10).

Univariate analysis of the alkali database identified that 1 VOC increased and 1 decreased between group “days 0–21” versus “days 21+” out of 33 VOCs (6%) (Table 4). 

Multivariate analysis performed on the alkali dataset via week categories identified nine VOCs as statistically significant (Table S11). In the alkali analysis, four VOCs increased towards death, three decreased and two were significant without a clear trend towards death (Table S11). However, post hoc analysis did not consistently show any significance between the different week group categories.

Analysis of the alkali dataset by linear regression analysis identified that one VOC increased and two VOCs decreased towards death. Although, two other VOCs, one increasing and one decreasing, approached significance (*p* = 0.065 and *p* = 0.052, respectively) (Table 4).

Combining univariate and linear regression of the identified VOCs showed that two VOCs increased and three VOCs decreased in the last weeks of life (Table 4).

**Figure 5:**
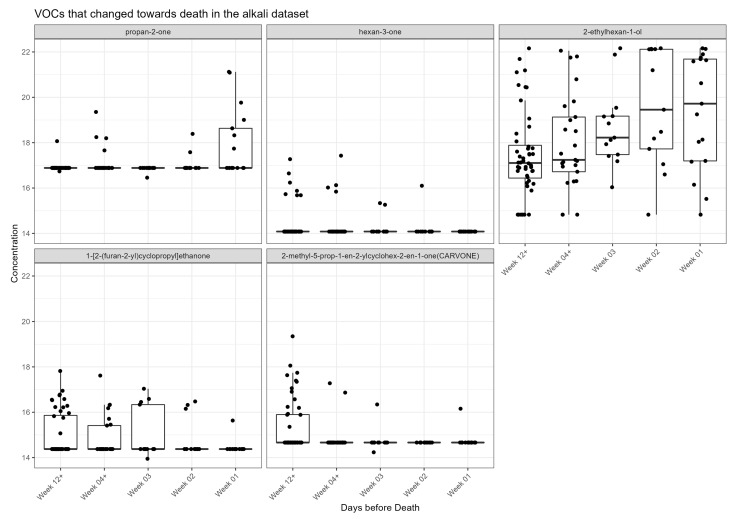
VOCs that change towards death in the alkali dataset. Jitter box plot graph for each metabolite identified as significant from univariate and linear regression analysis. The centre line is the median; box limits are upper and lower quartiles; whiskers are 1.5× interquartile range; points are individual observations. *Y* axis is VOC concentration on glog scale.

Visual inspection of the box plots of each VOC by week categories towards death showed two (alkali) increased towards death, whereas three decreased towards death (Figure 5).

In the alkalinised set, the two VOCs found to increase towards death were a ketone and an alcohol. The three others decreased towards death in the alkali dataset were a ketone, hydrocarbon and one was unclassified by all libraries.

Alkali dataset analysis by Cox Lasso did not identify a statistically significant model.

### 2.3. Comparing Acid and Alkali Datasets

Only two VOCs were found by both acid and alkali methods to change with significance towards death: propan-2-one, which increased, and 2-methyl-5-prop-1-en-2-ylcyclohex-2-en-1-one (Carvone), which decreased. Neither of these had CV >35% in either the acid or alkali pool (Tables S3 and S10).

## 3. Discussion

In this study, our findings provide evidence to support the hypothesis that there are specific metabolic changes associated with the dying process in cancer. Headspace GC-MS analysis of urine from patients with lung cancer identified that 12 VOCs increased and 25 VOCs decreased within the last 3 weeks of life. These VOCs are potential biomarkers of dying in lung cancer. A Cox Lasso regression analysis model using eight VOCs predicted dying with an AUC of 0.77 at 30 days, 0.78 at 20 days and 0.85 at 10 days.

Five papers have reported urinary VOCs in lung cancer and named 51 identified VOCs as potential biomarkers of cancer [26,27,28,29,30] (Table S12). It is interesting to note that the five VOCs we found increased towards death were also reported as potential biomarkers of lung cancer. In the acid dataset, they were heptan-2-one [26,27,28,30], propan-2-one [28], cyclohexanone [29] and nonan-2-one [26]. In our alkali dataset, 2-ethylhexan-1-ol [26,27] increased towards death. In addition, 1-methyl-4-propan-2-yl-7-oxabicyclo[2.2.1]heptane, which we found decreased toward death, was reported as being able to discriminate healthy control from lung cancer [28]. Five of these six VOCs had CV <35%.

One paper, which aimed to use VOCs to diagnose lung cancer at an early stage, demonstrated that the eight VOCs they discovered had increasing abundance of these VOCs with each stage of the cancer [30]. Of these, heptan-2-one was present in our acid dataset. This raises the possibility that our VOCs indicate increased disease burden towards the end of life. However, without collecting urine samples alongside staging scans, which are not routinely performed on a patient considered to be at the end of their life, definitive proof of this will be difficult to obtain. Given that only one of our VOCs has been shown to increase with stage of disease, it also raises the possibility that metabolic processes other than increased disease burden may contribute to the dying process. Little is known about the 14 VOCs that increase in the last weeks of life despite searching the PubChem, HMDB and Cancer Odor Databases (COD) [31,32] (Tables S13 and S14). Propan-2-one (acetone) is formed from fatty acid oxidation, which happens during fasting, diabetic ketoacidosis and strenuous exercise, and occurs almost entirely within the liver and to a smaller extent in the lung and kidney [33]. Acetone’s increase towards death in our data may be due to decreased oral intake towards the end of life. Benzaldehyde is involved in several metabolic pathways, e.g., glycolysis/gluconeogenesis, tryptophan metabolism and fatty acid metabolism [34], but it appears unstable in our analysis CV >35%, and does not have a clear trend in our dataset.

Some of the VOCs had previously been described in other biospecimens. Propan-2-one, heptan-2-one, cyclohexanone and phenol were previously detected in urine from patients with gastro-oesophageal [35] and lung cancer [27,29]. Propan-2-one, heptan-2-one, cyclohexanone, phenol and benzaldehyde were found in the breath of patients with breast [36], thyroid [37], colorectal cancer [38], gastro-oesophageal [39,40,41] and lung cancers [39,42,43,44,45,46]. Phenol was found in urine of patients with colorectal [47], bladder and breast cancers [48,49].

It is noteworthy that of some of these VOCs described in other cancers, many share a similar underlying biology to lung cancer. However other diseases implicated in these VOCs were wide-ranging, such as (E)-non-3-en-2-one, implicated in non-alcoholic fatty liver disease, Alzheimer’s disease, Parkinson’s disease [50] and diabetes mellitus type 2 [51].

Of the eight VOCs used in the Cox Lasso regression model, propan-2-one, as mentioned previously, is formed from fatty acid oxidation. Eucalyptol is used in flavourings, fragrances and cosmetics and 3,7-dimethyl-3-octanol is another flavouring. Their decrease may be associated with decreased oral intake towards the end of life. Little is known about the other VOCs in this model. Validation in an independent cohort of this model is required.

We believe our cohort of patients was representative of what you might expect of a cohort with lung cancer. The median age was 70, which matched Cancer Research UK’s data lung cancer diagnosis peak at age 70–74 [52]. Approximately 50% had underlying chronic obstructive pulmonary disease (COPD), estimated to affect 40–70% of lung cancer patients [53]. Around 9% of cases had depression, in keeping with the 8.7% prevalence among adults aged 65 and over [54]. Approximately 10% of our patients had chronic kidney disease (CKD), which has a prevalence of 12.0–14.5% (CKD stages 3–5) in those aged 65–74 years [55]. A total of 6% of our cohort had heart failure, in keeping with 4.3% among persons aged 65 to 70 years old in 2012 [56,57]. Diabetes was present in approximately 15%, slightly higher than the national average of 7% [58].

There are limitations to our study in relation to sample collection, storage and processing. The aim was to collect a 5 mL urine sample in a glass container twice a week, if possible, per participant. However, research nurse availability and the nature of working with patients towards their end of life meant this was not always feasible. Therefore, longitudinal analysis of VOCs from all patient was not possible. In order not to bias the results, only one sample per patient (the one closest to death) was included in the analysis.

Samples were collected in 10 mL glass vials. It is known that vials play a significant role in analytical analysis and result reproducibility. Therefore, vials must be inert and free of extractables or leachables to prevent affecting results [59]. We therefore collected urine in the same inert glass vials that were suitable for running on the GC-MS. Since ice expands on freezing, nurses were encouraged to fill the 10 mL vial approximately half full, to prevent the vial cracking on freezing. This meant that sample volume was limited and thus a direct comparison and acid and alkali datasets were not possible. However, it is clear that the acid dataset led to the detection of more VOCs and more VOCs which changed towards death.

There was variation in the time between sample collection and freezing, this varied between 0 to 3 h. However, most were frozen within the hour. This is in keeping with recommendations that ‘urine intended for VOC analysis should be frozen within 12 h of voiding to prevent excessive sample degradation and loss of signal’ [60]. Future work must therefore explore the stability of the VOCs identified as changing towards death.

Whilst samples were collected between June 2016 and September 2018, they were not prepared and run on the GC-MS until October 2019. This meant that it was possible to run all samples as a batch in this biomarker discovery project. Running samples as a batch minimises alterations in GC-MS. There is no evidence that storage at −20 °C has a negative influence on the presence of VOCs in headspace gases from urine samples [13,61]. One paper demonstrated that all analytes were stable in urine samples stored at −20 °C for a week, but, significantly, most analytes in urine samples are stable even up to 10 freeze–thaw cycles [62]. Whilst our samples were stored for over 3 years in some instances, all samples were collected, stored and analysed randomly.

The very nature of VOCs mean they are often present in one sample, but not another. During our analysis, we excluded VOCs that were not present in greater than 20% of any one “week” group. Further work is required to confirm the identity all these compounds. In particular, in the Cox Lasso model, one compound could be either nonan-2-one or 5-methylhexan-2-one, whilst another might be 3,7-dimethyloctan-3-ol or 3-methylpentan-3-ol.

The CV of some of the VOCs we reported was greater than 40%. We have been careful to acknowledge these. We suspect their wide CV is due to (1) being close to the limit of detection and (2) the homogeneity of the pooled sample. Three VOCs have no CV in the pooled samples; these were infrequently detected, but present in greater >20% in any one week category. We hypothesise that their abundance in the pool would have been diluted to below the limit of detection. Future work ought to use samples pooled by week categories. Further work should determine the limits of detection of key VOCs in our system.

The choice of SPME fibre limits the chemical window you are analysing. There are several commercially available coatings for SPME, and each has its own merits and, therefore, applications. We have reported that CAR/DVB/PDMS SPME fibre sorbs significantly more VOCs than from a CAR-PDMS fibre [20]. We selected CAR/DVB/PDMS because it has a wide mass range (C3-C30) and wide polarity (polar, non-polar and amines); this SPME coating is sometimes referred to as bipolar and is suitable for detecting odours and flavours [19]. However, using another type of SPME fibre may yield other results [63]; indeed, one paper showed that using a Needle Trap Device (NTD) with a triple bed DVB/CarX/Car1000 allowed a range of big and polar volatiles in contrast to the small and less polar volatiles to be adsorbed [28]. Other methods of detecting some of these VOCs should be investigated. We have only used HS-SPME-GC-MS. Alternatively, another method of GC-MS or other technology, such as a colorimetric sensor array [64] or gas chromatography-sensor system [10,65], might yield complementary results.

Predicting when a patient with advanced cancer is likely to die is a challenge and currently no prognostic biomarker is available [2]. Several validated prognostic tools aim to predict survival of patients with advanced cancer [5]. A recent comparison of five validated prognostic tools showed the best were only as accurate as expert clinician judgement; the overall accuracy was 61% [6,7,66]. Accurate prognostic information is essential to co-ordinate and manage care in response to need, whilst avoiding burdensome and unnecessary interventions. The early recognition that a person may be dying is central to all the priorities for improving peoples’ experience of care in the last days and hours of life. This is the first study to use a metabolomics approach to investigate the dying process in the last weeks of life.

Using HS-SPME-GC-MS analysis of urine, which provides a non-invasive matrix, we discovered VOCs that could be potential prognostic biomarkers for patients dying with lung cancer. These VOCs could be used as a tool to provide additional prognostic information to help inform expert clinician judgement and subsequent decision making.

## 4. Materials and Methods

### 4.1. Setting, Patient Recruitment and Ethical Consent

The study was conducted at 6 hospital and hospice sites in the North West of England from June 2016 to September 2018. The six sites were as follows: Aintree Hospital, Royal Liverpool Hospital, The Clatterbridge Cancer Centre, Marie Curie Hospice, Liverpool, Whiston Hospital and Willowbrook Hospice. Ethical approval was provided by North Wales (West) Research Ethics Committee (REC reference 15/WA/0464). Participants were enrolled in the study as previously described [67]. All participants had incurable disease. Participant clinical characteristics were collected. TNM staging data were not collected in this study as these are used at cancer diagnosis to predict 5-year survival and guide clinician treatment.

### 4.2. GCMS Methods

#### 4.2.1. Urine Samples for GCMS VOC Analysis

Each participant donated a 5 mL urine sample in a glass container twice a week, if possible. For those participants with a urinary catheter (*n* = 17 in acid dataset, *n* = 15 in alkali dataset), the urine was collected using a sterile needle and syringe from the catheter port. The samples were stored in freezers on site, at −20 °C at most sites and −80 °C at one site in 10 mL glass vials (vials, SU860100 and screw caps SU860101, Supelco from Merck, Dorset, UK). It is known that vials play a significant role in analytical analysis and result reproducibility. Therefore, vials must be inert and free of extractables or leachables to prevent affecting results [59]. We therefore collected urine in the same inert glass vials that were suitable for running on the GC-MS. Time between sample collection and freezing varied between almost immediately and up to 3 h, most within the hour. Samples were transferred to the University of Liverpool and stored at −20 °C The frozen urine was thawed at room temperature for 1–3 h and divided into 1 mL aliquots in 10 mL headspace vials with magnetic screw caps (vials, SU860100 and screw caps SU860101, Supelco from Merck, Dorset, UK). The aliquots were then refrozen and stored prior to sample preparation.

#### 4.2.2. Urine Sample Preparation

An amount of 1 mL of urine was either treated with acid or alkali as previously described [24], i.e., 1 mL of defrosted urine was treated with either 0.2 mL of 5 M sulphuric acid solution (H2SO4) (#12963634, Fisher Scientific, Loughborough) or 5 M sodium hydroxide (NaOH) solution (S8263-150ML, Sigma Aldrich, Dorset, UK), and vortexed, ready for HS-SMPE-GC-MS analysis. All samples were prepared and run on the GC-MS in a 4-week period in October 2019. There were between 8 and 17 samples, with at least 1 blank, 1 lab air and 1 pooled sample each day.

#### 4.2.3. Headspace-SPME-GC-MS Analysis

A Clarus 500 GC-MS quadruple bench top system (Perkin Elmer, Beaconsfield, UK) was used in combination with a Combi PAL autosampler (CTC Analytics, Zwingen, Switzerland). The GC column used was a Zebron ZB-624 with inner diameter 0.25 mm, length 60 m and film thickness 1.4 μm (Phenomenex, Macclesfield, UK). The carrier gas used was helium of 99.996% purity (BOC, Sheffield, UK). A divinylbenzene/carboxen/polydimethylsiloxane (DVB/CAR/PDMS) SPME fibre (needle size 23 ga, StableFlex, for use with autosampler (Sigma Aldrich)) was preconditioned before use. There are several commercially available coatings for SPME, and each has its own merits and therefore applications. We have reported that CAR/DVB/PDMS SPME fibre sorbs significantly more VOCs than from a CAR-PDMS fibre [20]. We selected CAR/DVB/PDMS because it has a wide mass range (C3-C30) and wide polarity (polar, non-polar and amines); this SPME coating is sometimes referred to as bipolar and is suitable for detecting odours and flavours [19]. Urine samples were incubated at 60 °C for 30 min, followed by the extraction of volatiles from the headspace of the vial and adsorption to the SPME fibre. The fibre was then inserted into the GC injection port for desorption at 220 °C for 5 min. The initial temperature of the GC oven was set at 40 °C and held for 2 min before increasing to 220 °C at a rate of 5 °C/min and then held for 4 min, with a total run time of 42 min. A solvent delay was set for the first 4 min and the MS was operated in positive electron impact ionisation EI+ mode, scanning from ion mass fragments 10–300 m/z, with an interscan delay of 0.1 s and a resolution of 1000 at FWHM (Full Width at Half Maximum). The helium gas flow rate was set at 1 mL/min. All samples were randomly injected. These conditions were the same as Aggio et al. 2016 [68].

#### 4.2.4. System Suitability and Quality Control

To warrant for potential contaminants or fluctuations in GC-MS measurement, we used a set of quality controls processed under the same experimental conditions as ‘real’ samples. ‘Blank’ samples were capped empty vials and run after every eight real samples. In addition, a ‘laboratory air’ (uncapped empty vial, where the SPME fibre sat and sampled the air for 20 min at room temperature) was run before every batch.

The analytical sequence of acid- and alkaline-treated urine samples was designed according to published guidance [69]. We produced a set of QC samples by making a single pooled sample from a representative subset (n = 50) of real urine samples in our study. We then prepared 1 mL aliquots from that pooled sample. One pooled QC urine sample aliquot treated with acid or alkali was run through the GC-MS with each batch of real samples, these acted as technical replicates and as a measurement of systemic stability of the SPME-GC-MS over time. The pooled samples confirmed the system was stable (Figure S6).

#### 4.2.5. GC-MS VOC Library Building and Data Analysis

After SPME-GC-MS, chromatograms were analysed for individual peaks by the computer software Automated Mass Spectral Deconvolution and Identification System (AMDIS) (version 2.71), in conjunction with the National Institute of Standards and Technology (NIST) mass spectral library software (version 17). Peaks were added to the library if there was a forward match greater than 800/1000. An acid-alkali urine library was built by examining all the pooled QC samples run and 10% of the ‘real’ samples per treatment group. The final library contained 173 unique VOCs, as previously published. A batch report was generated from AMDIS using our library with deconvolution settings as follows: component width of 10, adjacent peak subtraction of one, low resolution, low sensitivity and high shape requirements. R package Metab was used to generate a list of VOCs per sample, using a half a minute time window [70]. Compounds found in either lab air or blank samples were thought to be possible contaminants if they were present in >50% of all control samples; their removal from statistical analysis ensured that VOCs originated from urine samples being tested and prevented carry-over of VOCs on the SPME fibre between samples. Contaminants identified with quality control samples were removed before statistical analysis [24]. A total of 13 silane contaminants, ethanol and 2-nitrobenzene-1,4-dicarboxamide were removed from the analysis (Table S15). Since some chemicals could be in both the lab environment and in urine, compounds found in either lab air or blank samples were thought to be possible contaminants if they were present in >50% of all control sample.

VOCs were classified as published previously [24] by adapting the MeSH (Medical Subject Headings) database, which is found on the PubChem website [71].

#### 4.2.6. Statistical Analysis

Statistical analysis was performed using R software [72], version 4.2.1. Base R was used for most of the basic statistical analysis and the ggplot2 package was used for visualisation. VOCs found in <20% of samples in any one “Week” category were removed. Week categories were as follows: Week 01 = days 0–7 before death; Week 02 = days 8–14 before death; Week 03 = days 15–21 before death; Week 04+ = days 22–84 before death; Week 12+ = days 84+ before death. The remaining missing values were imputed by replacing the missing values with half of the minimum value for that VOC [73]. When numerous samples are analysed and some appear to have missing data, it is thought it is unlikely to mean there are no data, rather the VOC was undetected on that occasion. A number of metabolomics studies have been undertaken to determine how to deal with such data. There is no correct answer [74], but we have chosen to replace the missing values (NA) with a value that is half that of the minimum of the rest of the set of samples (Other groups replace with an arbitrary number, often 1, or use KNN to determine an alternative replacement [75].

Data were normalised by probabilistic quotient normalisation (PQN) and glog transformed. During PQN, each sample is normalised to a reference sample, using dilution factors. The reference sample is a conceptual one calculated from the median of all the samples [76]. PQN is a robust method to account for different dilution effects of biofluids. This method is based on the calculation of a most probable dilution factor (median) by looking at the distribution of the quotients of the amplitudes of a test spectrum by those of a reference spectrum. The glog transformation method was based on previous metabolomic data [77]. Although multiple samples were collected from patients, only the final sample was included in the analysis. Unequal variance was assumed.

For univariate analysis: We first considered whether there were differences in VOC abundance between samples collected within 21 days of death and those collected further from death. Each VOC was considered separately. The VOC fold changes were calculated from the dividing the means of each group. The mean used had been transformed back to the original scale by inverting the glog. We used T-Tests, assuming unequal variance and the Benjamini–Hochberg correction for multiple testing [61] using RStudio version 2021.09.0 [72]. Those with fold change (FC > 1.2, FC < −1.2) and *p* < 0.05 BH-adjusted were considered significant.

As a sensitivity analysis, to investigate the choice of time before death boundary, we also repeated these tests with boundaries of 7, 14 and 28 days (Table S4).

We next assessed whether VOC abundances changed in the weeks leading up to death, comparing abundances in five time categories. Analysis of Variance (ANOVA) (Welch test) was performed using RStudio version 2021.09.0 using the function oneway.test [72]. Week categories were as follows: Week 01 = days 0–7 before death; Week 02 = days 8–14 before death; Week 03 = days 15–21 before death; Week 04+ = days 22–84 before death; Week 12+ = days 84+ before death. Post hoc comparisons of each time category were calculated using pairwise *t*-tests. *p*-values were adjusted by Benjamini–Hochberg correction [78].

Since changes over time in VOC abundance were sometimes evident, linear regression models were used to assess the change in VOC abundance in the weeks before death. This was performed using lm() function in R. We censored observations greater 21 days before death. VOC abundance was considered as the dependent variable (y value) and time before death as the independent variable (x value). Censoring at 21 days meant any timepoint more than 21 days before death was considered to be 21. As with the univariate analysis, we present the results censored at 21 days, and additional linear regression models with censoring times before death are included in Table S6. *p*-values were calculated assuming heteroscedasticity and were adjusted by Benjamini–Hochberg correction [78].

Data visualisation: data were visualised using the ggplot2 package on R version 4.2.1 and RStudio version 2021.09.0 [72]. Volcano plots were plotted using R.

#### 4.2.7. Cox Lasso Prediction Modelling

A Cox proportional hazards model with Lasso penalty to derive a prediction model was used for assessing the last days of life in our cohort [79]. Cox models are commonly used to assess factors associated with time to death [25]. Cox models account for censoring in the data (the fact that time of death is not observed for everyone since it is beyond the observation period). The Lasso regression models have been developed in order to identify the most strongly associated predictors of an outcome. The model includes a penalty term, which penalises a model with too many predictors, thus including only the most important predictors. Lasso methods are considered more robust than model selection methods when there are large numbers of predictors (in our case, VOC abundances) [80]. Cox Lasso models are similar to the standard Cox model but shrink parameter estimates towards zero, reducing over-fitting due to the large number of potential metabolites being considered as possible predictors of death. Administrative censoring was applied if the individual was still alive 100 days after their sample was supplied. In these models, the time to death is considered as the outcome. The abundances of all VOCs are considered as possible predictors, and the lasso approach identifies the VOCs most strongly associated with time to death.

A penalty parameter (lambda) was imposed to determine the amount of smoothing chosen when 10-fold cross validation was performed. The value of lambda that gave minimum mean cross-validated error was used for both the prediction model and internal validation.

To guard against overoptimistic assessment of the predictive accuracy of our model, the model was internally validated using bootstrap resampling with 1000 iterations. The penalty parameter was fixed from the original Cox Lasso model to fit to the whole dataset, and then, for each bootstrap sample, a Cox Lasso model was fitted and time-dependent area under the curve was calculated [81]. Model calibration was assessed with each bootstrap sample by comparing the observed and expected survival probabilities, splitting the predicted risks into three groups (denoted low/medium/high survival). Calibration was performed at 14, 21 and 28 days. Kaplan–Meier curves were used to visualise the survival probabilities based on 21-day predicted risk. Log-rank tests were used to statistically compare the survival curves. Analysis was performed in R Studio version 1.4.1717 and used the packages “glmnet”, “survival” and “hdnom” [72].

## 5. Conclusions

In this study, we hypothesised that a dying process from cancer is associated with metabolic changes and specifically with changes in VOCs. Headspace GC-MS analysis of urine from patients with lung cancer identified that 12 VOCs increased and 25 VOCs decreased within the last 3 weeks of life. These are potential biomarkers of dying in lung cancer.

## 6. Patents

The results from our work were submitted for a patent. UK Patent Application No GB2204213.9; Biology of dying; The University of Liverpool.

## Figures and Tables

**Table 1:** Clinical Characteristics of the Patients included in this study.

	**Acid Dataset**	**Alkali Dataset**
**Total samples run on GC-MS**	n = 205	n = 150
**Samples closest to death, 1 sample/patient**	n = 144	n = 116
**Patients**	**Absolute** **number /144 (%)**	**Absolute number** **/116 (%)**
**Sex**		
Female:male	70:74 (49:51)	53:63 (46:54)
**Diagnosis**		
NSCLC † (Adenocarcinoma)	54 (38)	43 (37)
NSCLC † (Squamous)	29 (20)	23 (20)
NSCLC † (Large Cell)	1 (1)	1 (1)
SCLC ‡	28 (19)	24 (21)
Radiological or Clinical Diagnosis §	31 (22)	24 (21)
Mesothelioma	1 (1)	1 (1)
**Age (years)**		
**Median (range)**	70.50 (47–90)	70.00 (47–90)
40–49	4 (3)	3 (3)
50–59	22 (15)	16 (14)
60–69	46 (32)	41 (35)
70–79	43 (30)	31 (27)
80–89	29 (20)	24 (21)
90–100	0 (0)	1 (1)
**Ethnicity**		
Mixed—White and Black African	1 (1)	0 (0)
White—British	141 (98)	115 (99)
White—Irish	2 (1)	1 (1)
**Smoking status**		
Ex-smoker	25 (17)	19 (16)
Current	87 (60)	71 (61)
Never	32 (22)	26 (22)
**Co-morbidities**		
Chronic Obstructive Pulmonary Disease	54 (38)	47 (41)
Chronic Kidney Disease	8 (6)	6 (5)
Heart failure	9 (6)	7 (6)
Depression	13 (9)	8 (7)
Diabetes Mellitus	21 (15)	18 (16)
**Time of urine sample in relationship to death (time before death)**		
Day 0–21	55 (38)	43 (37)
Day 22+	89 (62)	73 (63)
**Time of urine sample in relationship to death (time before death)**		
Week 01	25 (17)	17 (15)
Week 02	16 (11)	13 (11)
Week 03	14 (10)	13 (11)
Week 04+	33 (23)	25 (22)
Week 12+	56 (39)	48 (41)

† NSCLC Non-small cell lung cancer; ‡ SCLC Small cell lung cancer; § Based on Multidisciplinary Team discussion.

**Table 2:** The 37 VOCs shown to change towards death by univariate analysis and linear regression analysis in the acid dataset.

		Univariate			Linear Regression	
RT	IUPAC Compound Name	BH pvals	Fold Change	Trend towards Death	Trend towards Death	padj Hetero
28.87	nonan-2-one or 5-methylhexan-2-one	0.005	1.757	↑	↑	0.000
21.71	heptan-2-one	0.012	1.913	↑	↑	0.008
25.35	Benzaldehyde *	0.018	2.152	↑		
32.67	(Z)-oct-2-enoic acid	0.038	1.370	↑	↑	0.015
31.31	5-ethyl-5-methyloxolan-2-one *	0.040	2.015	↑	↑	0.000
7.31	propan-2-one	0.042	1.382	↑	↑	0.015
24.67	2-methyl-5-methylsulfanylfuran *	0.044	1.863	↑	↑	0.017
23.69	3,4-dimethylhexan-2-one	0.044	1.523	↑	↑	0.015
19.65	hexane-3,4-dione **	0.044	1.255	↑	↑	0.046
22.81	Cyclohexanone **	0.051	1.848	↑	↑	0.006
27.76	Phenol *	0.056	2.261	↑		
27.57	4-methylpent-3-enoic acid				↑	0.000
37.09	1,2,4-triazole-3,4-diamine				↑	0.010
29.16	(E)-non-3-en-2-one **				↑	0.030
38.58	(E)-1-(2,6,6-trimethylcyclohexa-1,3-dien-1-yl)but-2-en-1-one	0.012	1.685	↓	↓	0.000
34.94	1,1,4a-trimethyl-4,5,6,7-tetrahydro-3H-naphthalen-2-one *	0.012	2.715	↓	↓	0.000
26.01	1-methyl-4-propan-2-ylbenzene (p-Cimene) *	0.018	2.044	↓	↓	0.001
28.37	5-(3,3-dimethyloxiran-2-yl)-3-methylpent-1-en-3-ol	0.030	1.935	↓	↓	0.000
37.32	2-buta-1,3-dienyl-1,3,5-trimethylbenzene *	0.038	2.139	↓	↓	0.000
34.49	(5R)-5-methyl-2-propan-2-ylidenecyclohexan-1-one-(Pulegone)	0.044	1.351	↓		
26.44	1,3,3-trimethyl-2-oxabicyclo[2.2.2]octane (Eucalyptol) *	0.044	2.009	↓	↓	0.031
35.9	2,6,6,10-tetramethyl-1-oxaspiro[4.5]dec-9-ene				↓	0.000
28.85	3,7-dimethyloctan-3-ol or 3-methylpentan-3-ol				↓	0.003
25.75	1-hydroperoxyhexane				↓	0.004
23.44	1-(furan-2-yl)ethenone *				↓	0.012
29.92	2-methoxyphenol *				↓	0.015
32.21	(1,4-dimethylpent-2-enyl)benzene				↓	0.015
26.89	1-methyl-4-propan-2-ylcyclohexa-1,4-diene				↓	0.015
30.94	1-methyl-4-prop-1-en-2-ylcyclohexa-1,3-diene or 1,2,4,5-tetramethylbenzene				↓	0.015
28.55	1-methyl-4-prop-1-en-2-ylbenzene or 1-methyl-2-prop-1-en-2-ylbenzene *				↓	0.016
34.77	2-methyl-5-prop-1-en-2-ylcyclohex-2-en-1-one (Carvone) *				↓	0.018
25.53	4,7,7-trimethylbicyclo[4.1.0]hept-2-ene((+)-4-Carene)				↓	0.022
25.59	1-methyl-4-propan-2-yl-7-oxabicyclo[2.2.1]heptane				↓	0.022
22.14	3,4-dimethylthiophene				↓	0.028
28.92	2-(5-ethenyl-5-methyloxolan-2-yl)propan-2-ol				↓	0.033
28.11	2,6-dimethyloct-7-en-2-ol				↓	0.045
32.14	5-methyl-2-propan-2-ylcyclohexan-1-ol (Menthol) *				↓	0.045

RT—Retention time. IUPAC—International Union of Pure and Applied Chemistry. BH pvals—Benjamini–Hochberg-adjusted *p* values. padj hetero—*p* value was calculated assuming heteroscedasticity and adjusted by Benjamini–Hochberg method. Table ranked by Trend towards Death, followed by BH *p* values of Univariate analysis in the first instance and then linear regression in the next instance. Trend towards Death, ↑ increased towards death. ↓ decreased towards death. Linear regression coefficients can be found in Table S6. * These 13 VOCs have CVs >40% (4 which increase towards death and 9 which decrease towards death). ** These three VOCs have no CV in pooled samples. Specific CVs can be found in Table S3.

**Table 3:** Cox Lasso Model.

	RT	IUPAC Compound Name	Coefficient in Cox Lasso Model
1	7.31	propan-2-one	0.205
2	19.65	hexane-3,4-dione **	0.009
3	26.44	1,3,3-trimethyl-2-oxabicyclo[2.2.2]octane(Eucalyptol) *	−0.038
4	28.85	3,7-dimethyloctan-3-ol or 3-methylpentan-3-ol	−0.029
5	28.87	nonan-2-one or 5-methylhexan-2-one	0.208
6	31.31	5-ethyl-5-methyloxolan-2-one *	0.007
7	34.94	1,1,4a-trimethyl-4,5,6,7-tetrahydro-3H-naphthalen-2-one *	−0.078
8	38.58	(E)-1-(2,6,6-trimethylcyclohexa-1,3-dien-1-yl)but-2-en-1-one	−0.015

VOCs included in the Cox Lasso model are included in the table, with their coefficients displayed. Colour coding demonstrates whether these VOCs increased (green) or decreased (red) towards death. The number of samples before death had 1 day added to them all, because the modelling could not work with samples from the day of death equal to zero in the model. Table ranked by retention time (RT). * These three VOCs have CVs >40%. ** This one VOC has no CV in pooled samples.

**Table 4:** Five VOCs shown to change towards death in alkali dataset, using univariate and linear regression analysis.

		Univariate			Linear Regression	
RT	IUPAC Compound Name	BH pvals	Fold Change	Trend towards Death	Trend towards Death	padj Hetero
26.93	2-ethylhexan-1-ol *	0.010	4.962	↑	↑	0.007
7.31	propan-2-one				↑	0.065
34.77	2-methyl-5-prop-1-en-2-ylcyclohex-2-en-1-one (Carvone)	0.016	0.630	↓	↓	0.007
33.25	1-[2-(furan-2-yl)cyclopropyl]ethanone				↓	0.016
17.5	hexan-3-one **				↓	0.052

RT—Retention time. IUPAC—International Union of Pure and Applied Chemistry. BH pvals—Benjamini–Hochberg-adjusted *p* values. padj hetero—*p* value was calculated assuming heteroscedasticity and adjusted by Benjamini–Hochberg method. Table ranked by Trend towards Death, followed by BH *p* values of Univariate analysis in the first instance and then linear regression in the next instance. Trend towards Death, ↑ increased towards death. ↓ decreased towards death. * This one VOC has CV >40% ** This VOC has no CV in pooled samples.

## Data Availability

Data is available in the Supplementary Materials.

## Supplementary Materials

The following supporting information can be downloaded at https://www.mdpi.com/article/10.3390/ijms24021591/s1. References [82,83,84,85,86,87,88,89,90,91,92,93,94,95] are cited in the supplementary materials.
