# Supplementary material for: GC-MS Techniques Investigating Potential Biomarkers of Dying in the Last Weeks with Lung Cancer
Source: Int J Mol Sci. 2023 Jan 13;24(2):1591. doi: 10.3390/ijms24021591 (PMC9867309; doi:10.3390/ijms24021591)
Supplement: Supplementary file 1 [file ijms-24-01591-s001.zip › GCMSPAPER_SupplementaryFigures_A4portrait_2022-12-27_2023-01-11.pptx]

## Slide 1
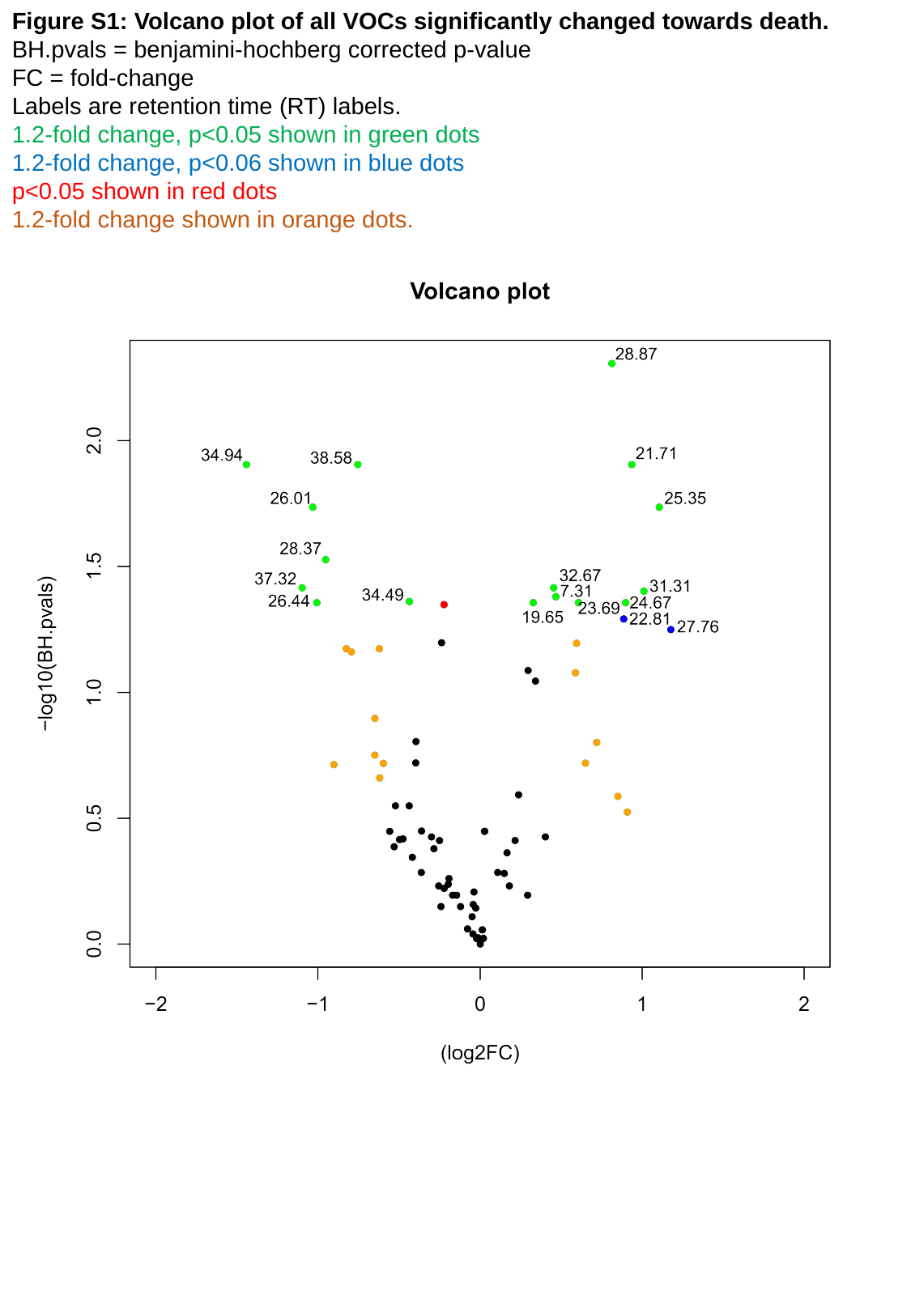

Figure S1: Volcano plot of all VOCs significantly changed towards death.
BH.pvals = benjamini-hochberg corrected p-value
FC = fold-change
Labels are retention time (RT) labels.
1.2-fold change, p<0.05 shown in green dots
1.2-fold change, p<0.06 shown in blue dots
p<0.05 shown in red dots
1.2-fold change shown in orange dots.

## Slide 2
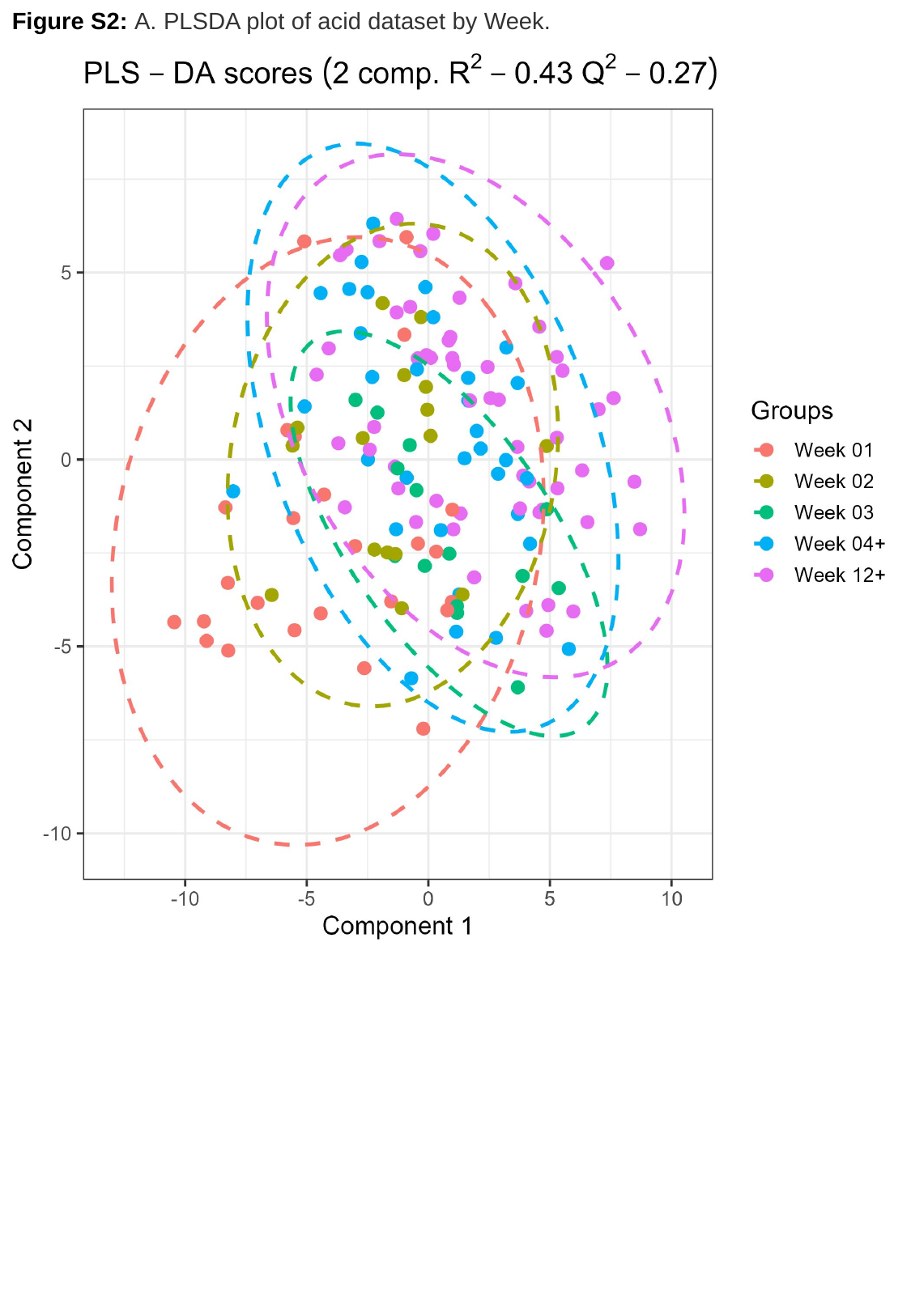

Figure S2: A. PLSDA plot of acid dataset by Week.

## Slide 3
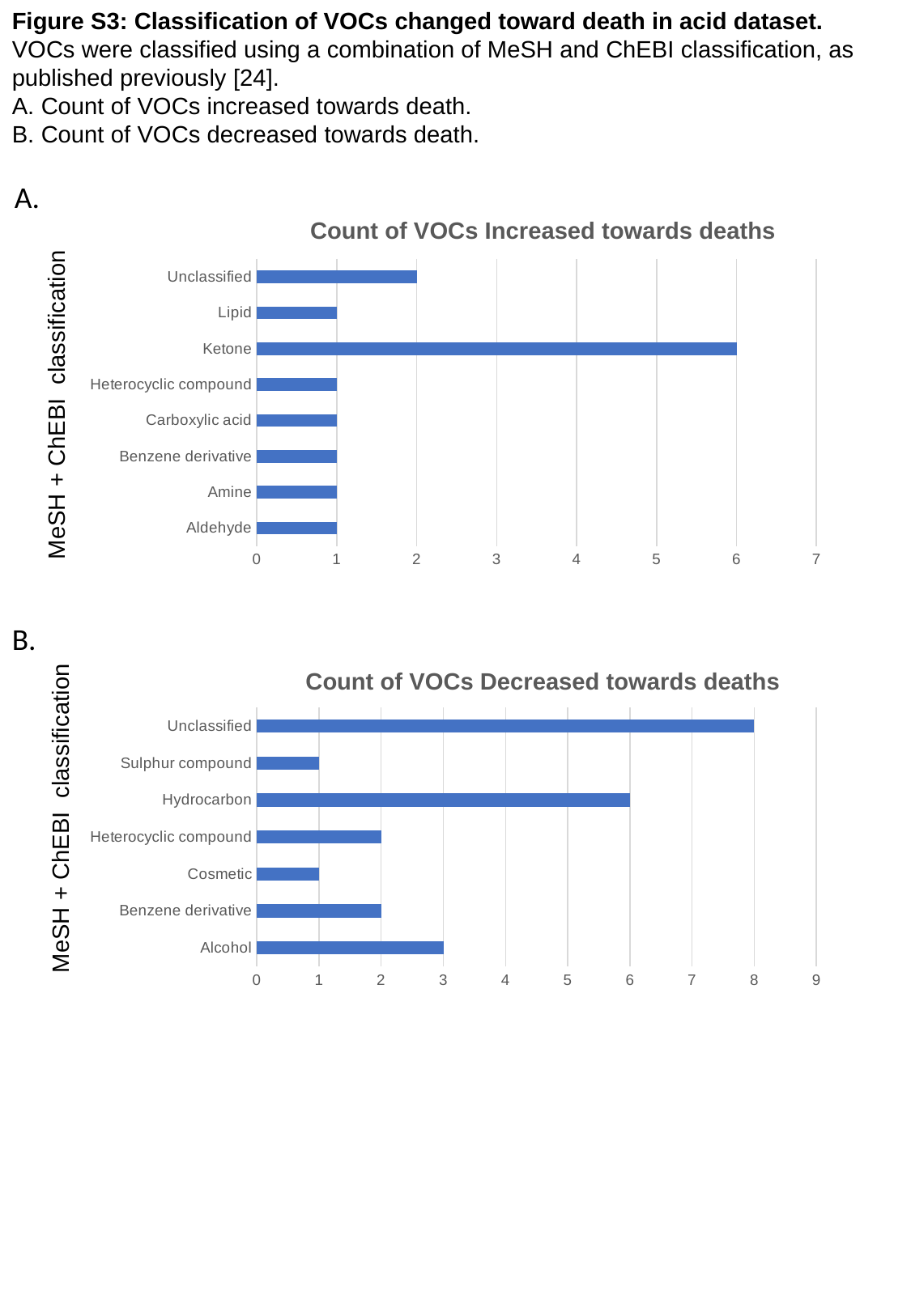

Figure S3: Classification of VOCs changed toward death in acid dataset.
VOCs were classified using a combination of MeSH and ChEBI classification, as published previously [24].
A. Count of VOCs increased towards death.
B. Count of VOCs decreased towards death.
A.
Count of VOCs Increased towards deaths
### Chart
| Category | Total |
|---|---|
| Aldehyde | 1.0 |
| Amine | 1.0 |
| Benzene derivative | 1.0 |
| Carboxylic acid | 1.0 |
| Heterocyclic compound | 1.0 |
| Ketone | 6.0 |
| Lipid | 1.0 |
| Unclassified | 2.0 |MeSH + ChEBI classification
B.
Count of VOCs Decreased towards deaths
### Chart
| Category | Total |
|---|---|
| Alcohol | 3.0 |
| Benzene derivative | 2.0 |
| Cosmetic | 1.0 |
| Heterocyclic compound | 2.0 |
| Hydrocarbon | 6.0 |
| Sulphur compound | 1.0 |
| Unclassified | 8.0 |MeSH + ChEBI classification

## Slide 4
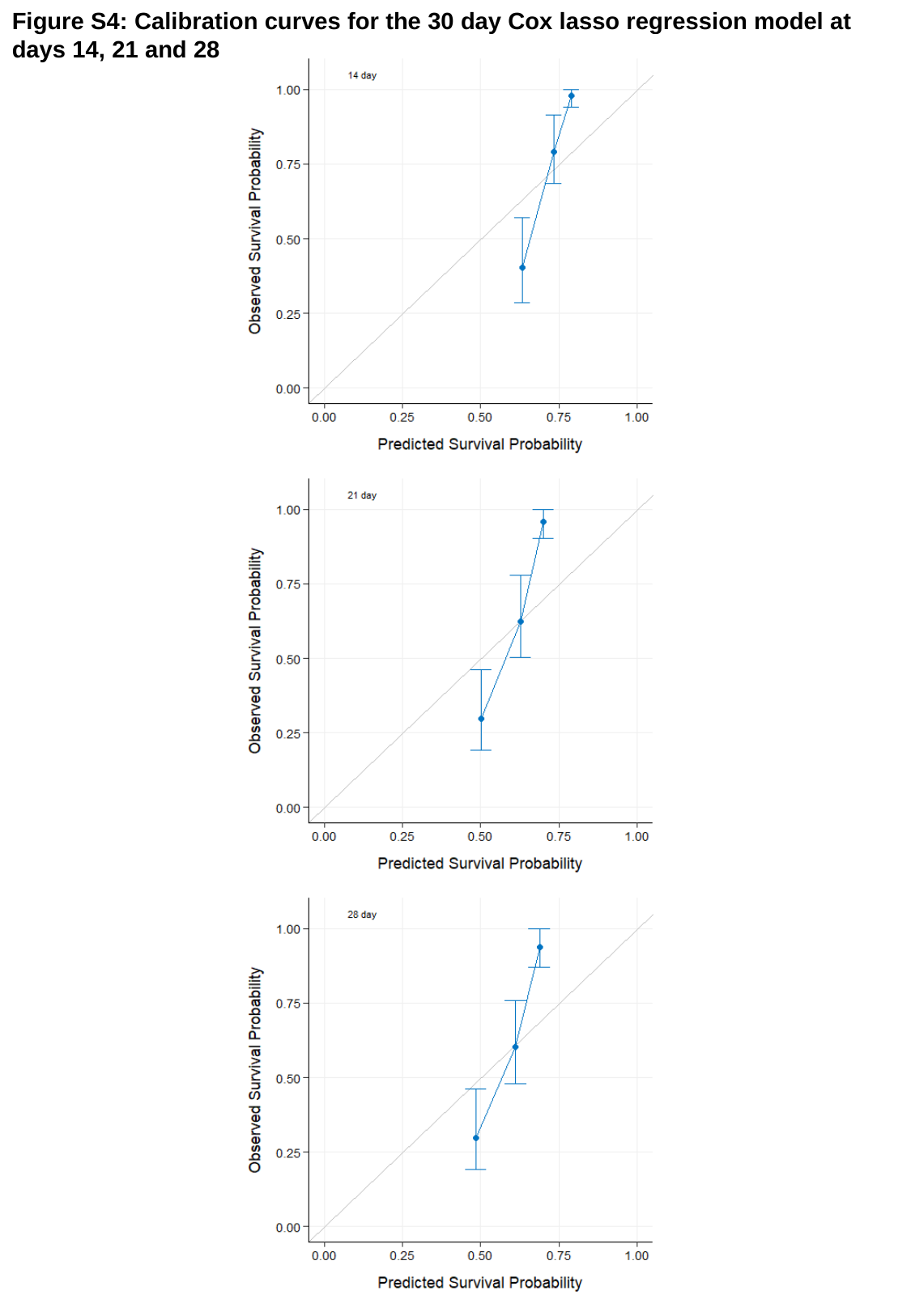

Figure S4: Calibration curves for the 30 day Cox lasso regression model at days 14, 21 and 28

## Slide 5
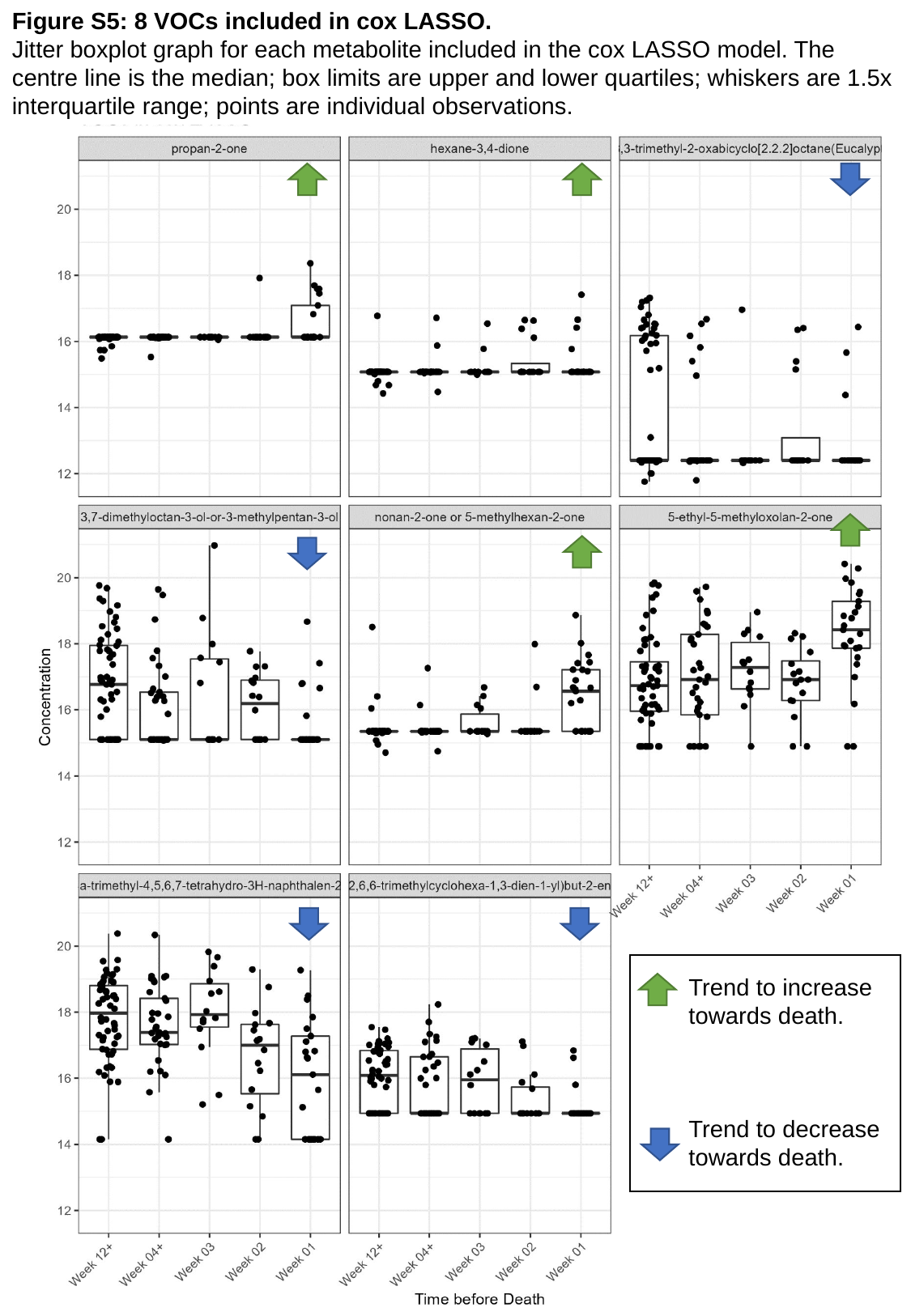

Figure S5: 8 VOCs included in cox LASSO.
Jitter boxplot graph for each metabolite included in the cox LASSO model. The centre line is the median; box limits are upper and lower quartiles; whiskers are 1.5x interquartile range; points are individual observations.
Trend to increase towards death.
Trend to decrease towards death.

## Slide 6
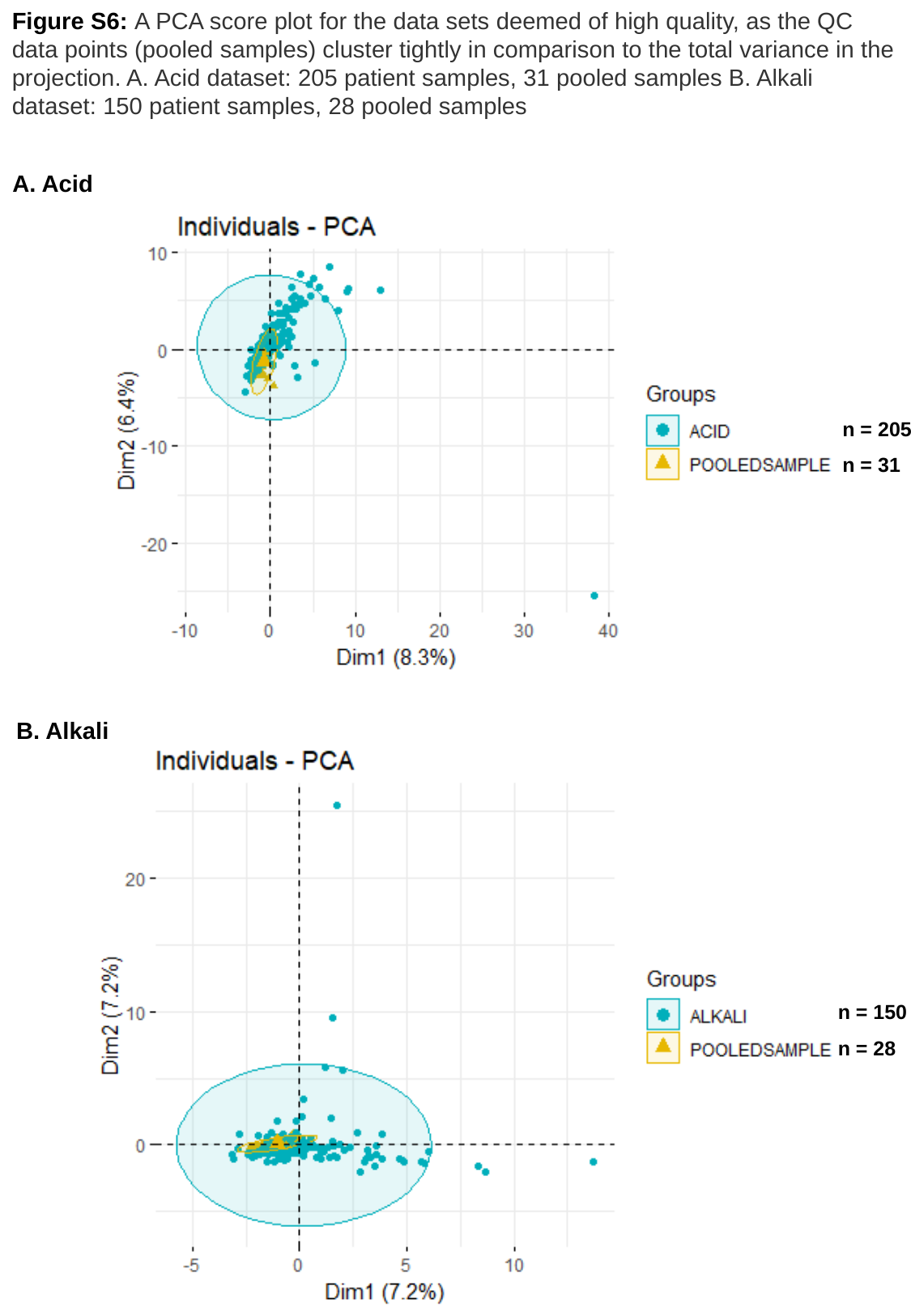

Figure S6: A PCA score plot for the data sets deemed of high quality, as the QC data points (pooled samples) cluster tightly in comparison to the total variance in the projection. A. Acid dataset: 205 patient samples, 31 pooled samples B. Alkali dataset: 150 patient samples, 28 pooled samples
A. Acid
n = 205
n = 31
B. Alkali
n = 150
n = 28
